# Association between Perinatal Outcomes and Maternal Risk Factors: A Cohort Study

**DOI:** 10.3390/medicina60071071

**Published:** 2024-06-29

**Authors:** Raquel Martin-Alonso, Paula Prieto, Irene Fernández-Buhigas, Cristina German-Fernandez, Cristina Aramburu, Victor Piqueras, Diana Cuenca-Gomez, Emilia Ferrer, Valeria Rolle, Belén Santacruz, María M. Gil

**Affiliations:** 1 Department of Obstetrics and Gynecology, Hospital Universitario de Torrejón, Torrejón de Ardoz, 28850 Madrid, Spain; rmartin@torrejonsalud.com (R.M.-A.); pprieto@torrejonsalud.com (P.P.);; 2 Faculty of Medicine, Universidad Francisco de Vitoria, Pozuelo de Alarcón, 28223 Madrid, Spain; 3 Statistics and Data Management Unit, iMaterna Foundation, Alcalá de Henares, 28806 Madrid, Spain; 4 Facultad de Estudios Estadísticos, Universidad Complutense de Madrid, 28040 Madrid, Spain

**Keywords:** cigarette smoking, body mass index, age, pregnancy, preeclampsia, diabetes, obesity, fetal, labor, birth weight

## Abstract

*Background and Objectives*: The aim of this study was to analyze the association between maternal risk factors, such as age, body mass index (BMI), and cigarette smoking, and perinatal outcomes. *Materials and Methods*: We conducted a retrospective analysis based on prospectively collected data at Hospital Universitario de Torrejón (Madrid, Spain) between September 2017 and December 2019. All pregnant women with singleton pregnancies and non-malformed live fetuses attending their routine ultrasound examination at 11+0 to 13+6 weeks’ gestation were invited to participate. The association between preeclampsia, preterm birth, gestational diabetes mellitus (GDM), small-for-gestational-age (SGA) or fetal-growth-restricted (FGR) neonates, and type of delivery and maternal age, BMI, and cigarette smoking was studied. Logistic mixed models were used to analyze the data. *Results*: A total of 1921 patients were included in the analysis. Women who were ≥40 years old had a significantly higher risk of having GDM (odds ratio (OR) 1.61, 95% confidence interval (CI) 1.08 to 2.36) and SGA neonates (OR 1.54, 95% CI 1.00 to 2.37). Women with a BMI < 18 had an increased rate of giving birth to SGA and FGR neonates (OR 3.28, 95% CI 1.51 to 7.05, and OR 3.73, 95% CI 1.54 to 8.37, respectively), whereas women with a BMI ≥ 35 had a higher risk of GDM (OR 3.10, 95% CI 1.95 to 4.89). Smoking increased the risk of having SGA and FGR neonates (OR 1.83, 95% CI 1.36 to 2.46, and OR 1.91, 95% CI 1.29 to 2.78). *Conclusions*: Advanced maternal age, low or high BMI, and smoking status are significant risk factors for pregnancy complications. Both clinicians and society should concentrate their efforts on addressing these factors to enhance reproductive health.

## 1. Introduction

Maternal characteristics such as age, body mass index (BMI), and cigarette smoking are important risk factors for pregnancy complications. Multiple studies have shown the association between these factors and adverse perinatal outcomes [1,2,3,4,5].

Many countries have reported a decline in birth rates, although the proportion of births in older women has increased [6,7]. Advanced maternal age (AMA), defined as pregnant women of 40 years and older, could be responsible for a substantial proportion of the increased rate of low-birth-weight (LBW) <2500 g, small-for-gestational-age (SGA), and preterm deliveries observed in the past decades [8,9,10]. There are also other complications that have been described in advanced-age mothers such as preeclampsia (PE) and gestational diabetes mellitus (GDM) [5,11,12,13]. A 2019 meta-analysis that studied the adverse perinatal outcomes related to advanced maternal age included 10 studies and concluded that women aged between 35 and 40 and older were more likely to present overweight, GDM, and gestational hypertension. Additionally, they were at a higher risk for adverse perinatal outcome such as preterm delivery or low-birth-weight babies [5]. It has also been reported that AMA mothers are more likely than younger women to experience labor dystocia [14] and cesarean delivery [10,11,15,16,17].

Obesity prevalence is increasing worldwide [18,19]. Maternal obesity carries significant risks and is likely to be associated with adverse perinatal outcomes such as GMD, gestational hypertension, PE, or large-for-gestational-age fetuses (LGA), and these risks appear to increase along with the severity of the condition [2,19,20,21]. Due to these obesity-related maternal disorders, obesity might increase the risk of medically indicated preterm birth, but whether obesity increases the risk for spontaneous preterm birth is still unknown [22]. Finally, obesity, has also been described as a risk factor for both programmed and intrapartum cesarean section [23,24]. The basis of many of these complications is likely to be related to the altered metabolic state associated with morbid obesity [25,26].

Despite the current obesity epidemic, maternal underweight remains a common but less well studied condition also with potential adverse perinatal outcomes [27]. Low maternal BMI at the beginning of pregnancy has been associated with preterm labor, LBW, SGA, fetal growth restriction (FGR), and cesarean section, with these risks increasing with the severity of the condition [3,22,27,28,29,30].

Smoking during pregnancy not only affects women’s own health but may also be associated with adverse perinatal and offspring outcomes, like preterm birth, LBW, SGA, and FGR [1,4,31], with a dose-dependent increase in risks [1]. Surprisingly, smoking during pregnancy has been associated with a reduced risk of preeclampsia [32,33].

In this study, we aimed to analyze the association between these three maternal risk factors—age, BMI and smoking—and adverse perinatal outcomes.

## 2. Materials and Methods

### 2.1. Study Design and Population

This is a retrospective analysis from prospectively collected data derived from a cohort study conducted to screen for preterm PE in a routine population [34]. All pregnant women with singleton pregnancies and non-malformed live fetuses attending their routine ultrasound examination at 11+0 to 13+6 weeks’ gestation at Hospital Universitario de Torrejón (Madrid, Spain) between September 2017 and December 2019 were invited to participate. The association between PE, preterm birth, GDM, SGA or FGR neonates, and type of delivery and BMI, maternal age, and smoking status at the beginning of the pregnancy was studied. This study was approved by the local Research Ethics Committee and all women provided written consent form.

During the 11+0 to 13+6 weeks hospital visit, patient characteristics and medical history were recorded in a clinical database (ViewPoint^®^ software version 5, GE Healthcare; Munich, Germany), including maternal age, race (White, Black, South Asian, East Asian, or Mixed), the method of conception (natural or using assisted reproductive technology defined as in vitro fertilization or use of ovulation drugs), smoking during pregnancy, weight, height (BMI was calculated as kg/m^2^), and medical and obstetric history. The obstetric history included parity (parous or nulliparous if no previous pregnancies at ≥24 weeks of gestation), and for parous women, previous PE, and gestational age at delivery of previous baby.

### 2.2. Pregnancy Outcomes

Participants were followed up according to the clinical protocols, and any pregnancy complications, as well as delivery data, were recorded by reviewing hospital/regional records or contacting delivering hospitals or the women’s general medical practitioners/midwives.

PE was diagnosed according to the American College of Obstetricians and Gynecologists [35]. GDM was diagnosed by means of a sequential model (O’Sullivan test and, if positive, a 100 mg Oral Glucose Tolerance Test (OGTT) according to the Diabetes in Pregnancy Spanish Group (Grupo Español de Diabetes y embarazo, GEDE) [36]. Preterm birth was defined as delivery before 37 weeks of gestation. Neonatal weight was assessed within the first 24 h of life and converted to centiles using the Fetal Medicine Foundation charts [37]. SGA was diagnosed when birth weight was <10th centile and FGR when birth weight was <3rd centile.

### 2.3. Statistical Analysis

Descriptive data were expressed as the median and interquartile range (IQR) and in proportions (absolute and relative frequencies). We studied the association of preterm birth, PE, GDM, fetal growth disorders (birth weight percentiles below the 10th, the 3rd, and above the 95th), and type of delivery with maternal age first (40 or more years compared to the group of less than 40), BMI second (35 or more and less than 18 compared to the group between 18 and 35) and smoking status third. For each variable of interest, we adjusted a multiple logistic regression model, ensuring at least 10 adverse outcomes per variable included in the development of each model. Adjusted odds ratios (aORs), their 95% confidence intervals (CIs), and *p*-values were computed. The level of significance was set at 0.05. All analyses were carried out with the statistical software R in its version 4.3.0 [38] and the packages table1 Version 1.4.3 [39] and sjPlot Package Version 2.8.14 [40].

## 3. Results

### 3.1. Study Population and Pregnancy Outcomes

We included 1921 patients in this analysis. Maternal characteristics according to risk factors are described in Table 1. Table 2 shows pregnancy outcomes according to maternal risk factors.

### 3.2. Risk Factors for Pregnancy Complications

#### 3.2.1. Maternal Age

There were 145 pregnant women who were 40 years old or older at the beginning of the pregnancy. After adjusting for possible confounders, this group of women showed a significantly higher risk of having GDM (aOR 1.61, 95% CI 1.08 to 2.36, *p* = 0.018), and SGA neonates (aOR 1.54, 95% CI 1.00 to 2.37, *p* = 0.049). However, no association was detected between maternal age ≥ 40 and preterm birth, mode of delivery, PE, or FGR (Table 3 and Table S1).

#### 3.2.2. Body Mass Index

Twenty-nine women had a BMI < 18 at the beginning of pregnancy. These women showed an increased rate of birth SGA (aOR 3.28, 95% CI 1.51 to 7.05, *p* = 0.002) and FGR neonates (aOR 3.73, 95% CI 1.54 to 8.37, *p* = 0.002). No significant differences were found in either group in the incidence of preterm delivery, mode of delivery, or PE (Table 3 and Table S2).

There were 90 women with a BMI ≥ 35. This group had a higher risk of GDM (aOR 3.10, 95% CI 1.95 to 4.89, *p* < 0.001) (Table 3). When using BMI as a continuous variable, the risk of GDM increases with increasing BMI (aOR 1.11, 95% CI 1.08 to 1.13, *p* < 0.001) (Table S3). In addition, there was an association between BMI ≥ 35 and fetal birth weight being >95th centile (aOR 3.50, 95% CI 1.37 to 8.91, *p* = 0.009) (Table 3 and Table S3). The risk of cesarean section was also increased (aOR 2.12, 95% CI 1.25 to 3.54).

#### 3.2.3. Smoking

There were 274 smokers at the beginning of the pregnancy. Pregnant smokers were at a higher risk of having SGA (aOR 1.83, 95% CI 1.36 to 2.46) and FGR (aOR 1.91, 95% CI 1.29 to 2.78) fetuses (Table 3 and Table S4). Smoking was not associated with preterm birth, mode of delivery, PE, or GDM.

## 4. Discussion

### 4.1. Main Findings of the Study

This study showed that, first, AMA and obesity are significant risk factors for GDM and, second, advanced maternal age, BMI < 18, and smoking at the beginning of pregnancy are risk factors for developing SGA and FGR fetuses.

### 4.2. Comparison with Previous Studies

Similar to previous studies, we identified AMA, the body mass index, and cigarette smoking as important maternal risk factors that must be considered while planning pregnancy care [1,2,3,4,5].

Women are postponing childbearing to their late 30s and beyond 40 around the world, but particularly in high-income countries [6,7]. In our cohort, 7.5% of pregnant women were 40 years old or more at the beginning of the pregnancy. Consistent with prior studies, our research confirms a higher incidence of GDM in older women [5,10,11,12]. This observation aligns with the well-established trend of a decrease in pancreatic β-cell function and insulin sensitivity with age [41,42]. As Cnattingius et al. and Khalil et al. [8,11] described in their studies, we also found an association between AMA and an increased risk of low birth weight. However, the underlying mechanism behind this association remains undetermined. Khalil et al. [11] carried out a retrospective study that included 76,158 singleton pregnancies. They concluded that not only is AMA a risk factor for GDM and SGA, but also for preeclampsia and cesarean section. In contrast, we found no evidence to establish an association between AMA and PE, nor with mode of delivery, although this might be due to our smaller sample size. Consistent with our findings, Khalil et al. also demonstrated no significant association between AMA and preterm delivery [11]. However, Pinheiro et al. [5] described in their meta-analysis an increased risk of preterm birth with increased maternal age. This inconsistency among the results could be explained by differences in the definition of preterm delivery, differentiation between spontaneous or iatrogenic preterm labor, and the baseline characteristics of the populations.

Obesity is a chronic disease, the prevalence of which is increasing worldwide, and is a major contributor to poor health and adverse perinatal outcomes [2,20,21,22,43]. In Spain, 10–15% of women of reproductive age are obese and around 20–29% are overweight [44]. As previously described [2,20,21,45,46,47,48], we found that high BMI is associated with a higher risk of GDM and LGA babies, although the latter was found not to be statistically significant after adjusting for other confounders. The association between maternal adiposity and LGA infants might be explained by fetal overnutrition, since an increased placental transfer of nutrients to the fetus might lead to an increased synthesis of insulin and insulin-like growth factors, both of which are growth-promoting hormones [49].

On the other hand, around 3.5% of the women in Spain are underweight, being more prevalent (between 5 and 10%) in women at reproductive age [44]. However, it remains a much less well-studied condition than obesity. In our sample, 1.6% of women had a BMI < 18.5, which is a much lower rate than expected from data published in previous studies [28,44]. Consistent with the existing literature, we found that maternal pre-pregnancy underweight was associated with an increased risk of LBW [3,22,27,28,29,30].

Interestingly, unlike most previous published studies [2,3,20,22,27,28,45], we did not find an association between extreme BMI and PE, preterm birth, or mode of delivery. These negative results could be related to a smaller than expected proportion of women with these conditions in our study.

Finally, smoking is a known risk factor for adverse perinatal outcomes including LBW, SGA, and FGR [4,31], which is consistent with our results. The mechanisms that could explain why maternal smoking may affect intrauterine growth and birth weight include vasoconstriction caused by nicotine (by inducing maternal catecholamine release), increased carboxyhaemoglobin levels in umbilical arteries which result in fetal hypoxia [50,51], or a decreased concentration of leptin [52]. On the other hand, we did not find any association between smoking and PE, which was also reported in a recent meta-analysis and systematic review [32,33]. In our study, no association was found between smoking and mode of delivery; however, Li et al. performed a retrospective cohort study with 20,477 (14, 6%) women who smoked during pregnancy and 119,396 controls that revealed that women who smoked were more likely to have a cesarean section for non-reassuring fetal status (adjusted odds ratio (OR) 1.16, 95% CI 1.07 to 1.26, *p* < 0.001) [53]. In contrast to previous studies [1,54,55], we did not find an association with preterm birth. Liu et al. [55] found that maternal smoking during either the first or second trimester of pregnancy was associated with an increased risk of preterm birth. These differences could be explained by the much smaller sample size of our study, as well as by differences in the maternal characteristics of the populations or in the number of cigarettes smoked per day that may contribute as confounders.

### 4.3. Clinical Implications

National efforts should prioritize raising awareness of modifiable risk factors before pregnancy, including maintaining healthy weight and promoting pregnancies at optimal maternal ages. Although AMA and increased BMI are not modifiable once gestation occurs, perinatal outcomes can still be improved by the early detection of pregnancy complications such as GDM and SGA.

On the other hand, smoking is a modifiable risk factor. Women of reproductive age or those who are pregnant and smoke should be strongly encouraged and supported to quit smoking before conception or during the early stages of pregnancy. Antenatal clinics should incorporate smoking cessation interventions, with heavy smokers receiving personalized counseling and follow-up tailored to their specific risks.

### 4.4. Strengths and Limitations

The main strength of this study relies on it being a prospective unselected cohort from a non-referral center, which is likely representative of the general population in our city.

However, its observational nature is a primary limitation, preventing the establishment of definite associations. Additionally, the limited number of cases for extreme ranges in all variables or for adverse perinatal outcomes may have hindered the identification of significant predictors.

## 5. Conclusions

Advanced maternal age, low or high BMI, and smoking status are significant risk factors for pregnancy complications. Both clinicians and society should concentrate their efforts on addressing these factors to enhance reproductive health.

## Figures and Tables

**Table 1:** Maternal characteristics of the study population according to risk factors.

	Overall (n = 1921)	Maternal Age		Body Mass Index			Smoker	
		Less Than 40 (n = 1776)	40 or more (n = 145)	<18 (n = 29)	18 to <35 (n = 1802)	≥35	No	Yes
						(n = 90)	(n = 1647)	(n = 274)
Maternal age in years,	33.6	33.1	41.2	29.8	33.7	32.8	33.8	32.3
median (IQR)	(30.0, 36.6)	(29.7, 35.9)	(40.5, 42.2)	(24.5, 33.9)	(30.1, 36.6)	(30, 36)	(30.3, 36.6)	(29.0, 36.0)
Body mass index in kg/m^2^, median (IQR)	24.0	23.9	24.9	17.2	23.9	38.0	24.0	24.4
	(21.7, 27.5)	(21.6, 27.5)	(22.2, 27.5)	(16.87, 17.5)	(21.7, 27)	(36.4, 40.3)	(21.7, 27.5)	(21.8, 27.6)
Smoker, n (%)	274 (14.3%)	259 (14.6%)	15 (10.3%)	6 (20.7%)	254 (14.1%)	14 (15.6%)	0	274 (100%)
Race, n (%)								
White	1873 (97.5%)	1735 (97.7%)	138 (95.2%)	29 (100%)	1756 (97.4%)	88 (97.8%)	1601 (97.2%)	272 (99.3%)
Black	30 (1.6%)	24 (1.4%)	6 (4.1%)	0	28 (1.6%)	2 (2.2%)	28 (1.7%)	2 (0.7%)
East Asian	9 (0.5%)	8 (0.5%)	1 (0.7%)	0	9 (0.5%)	0	9 (0.5%)	0
Mixed	5 (0.3%)	5 (0.3%)	0	0	5 (0.3%)	0	5 (0.3%)	0
South Asian	4 (0.2%)	4 (0.2%)	0	0	4 (0.2%)	0	4 (0.2%)	0
Nulliparity n (%)	847 (44.1%)	796 (44.8%)	51 (35.2%)	14 (48.3%)	802 (44.5%)	31 (34.4%)	713 (43.3%)	134 (48.9%)
Conception n (%)								
Spontaneous	1798 (93.6%)	1692 (95.3%)	106 (73.1%)	29 (100%)	1683 (93.4%)	86 (95.6%)	1539 (93.4%)	259 (94.5%)
Assisted reproductivetechniques	123 (6.4%)	84 (4.7%)	39 (26.9%)	0	119 (6.6%)	4 (4.4%)	108 (6.6%)	15 (5.5%)
Chronic hypertension n (%)	28 (1.5%)	22 (1.2%)	6 (4.1%)	0	22 (1.2%)	6 (6.7%)	25 (1.5%)	3 (1.1%)
Diabetes Mellitus n (%)								
Type 1	10 (0.5%)	9 (0.5%)	1 (0.7%)	0	9 (0.5%)	1 (1.1%)	10 (0.6%)	0
Type 2	5 (0.3%)	4 (0.2%)	1 (0.7%)	0	5 (0.3%)	0	4 (0.2%)	1 (0.4%)
APS and/or SLE n (%)	22 (1.1%)	20 (1.1%)	1 (1.4%)	0	22 (1.2%)	0	22 (1.3%)	0
Previous preeclampsia n (%)	55 (2.9%)	52 (2.9%)	3 (2.1%)	1 (3.2%)	49 (2.7%)	5 (5.6%)	49 (3.0%)	6 (2.2%)
Previous neonate’s birth weight <10th percentile n (%)	259 (13.5%)	234 (13.2%)	25 (17.2%)	5 (16.1%)	245(13.6%)	9(10%)	213 (12.9%)	46 (16.8%)
Previous neonate’s birth weight <3rd percentile n (%)	127 (6.6%)	112 (6.3%)	15 (10.3%)	4 (12.9%)	120 (6.7%)	3(3.3%)	102 (6.2%)	25 (9.1%)
Previous Gestational Diabetes n (%)	67 (3.5%)	57 (3.2%)	10 (6.9%)	0	57 (3.2%)	10 (11.1%)	56 (3.4%)	11 (4.0%)
Previous preterm birth n (%)	83 (4.3%)	74 (4.2%)	9 (6.2%)	1 (3.4%)	80 (4.4%)	2 (2.2%)	69 (4.2%)	14 (5.1%)
Previous fetal weight > 95% percentile n (%)	34 (3.2%)	32 (3.3%)	2 (2.1%)	0 (0%)	29 (2.9%)	5 (8.5%)	29 (3.1%)	5 (3.6%)

Results are expressed as the median (interquartile rage, IQR) and n and percentage (%) as required; APS: antiphospholipid syndrome; SLE: systemic lupus-erithematosus.

**Table 2:** Pregnancy outcomes according to maternal risk factors.

	Overall (n = 1921)	Maternal Age		Body Mass Index			Smoker	
		Less Than 40 (n = 1776)	40 or More (n = 145)	<18 (n = 29)	18 to 35 (n = 1802)	≥35 (n = 90)	No (n = 1647)	Yes (n = 274)
Neonatal outcome, n (%)								
Live birth	1916 (99.7%)	1771 (99.7%)	145 (100%)	29 (100%)	1797 (99.7%)	90 (100%)	1643 (99.8%)	273 (99.6%)
Neonatal death	2 (0.1%)	2 (0.1%)	0	0	2 (0.1%)	0	2 (0.1%)	0
Stillbirth	3 (0.2%)	3 (0.2%)	0	0	3 (0.2%)	0	2 (0.1%)	1 (0.4%)
Gestational age at birth in weeks, median (IQR)	39.0	39.0	39.0	39.0	39.0	39.0	39.0	39.0
	(38.0, 40.0)	(38.0, 40.0)	(38.0, 40.0)	(38.0, 40.0)	(38.0, 40.0)	(38.0, 40.0)	(38.0, 40.0)	(38.0, 40.0)
Preterm birth n (%)	118 (6.1%)	108 (6.1%)	10 (6.9%)	4 (13.8%)	108 (6.0%)	6 (6.7%)	104 (6.3%)	14 (5.1%)
Labor onset n (%)								
Spontaneous	967 (50.3%)	899 (50.6%)	68 (46.9%)	11 (37.9%)	927 (51.4%)	29 (32.2%)	840 (51.0%)	127 (46.4%)
Induced	817 (42.5%)	753 (42.4%)	64 (44.1%)	14 (48.3%)	749 (41.6%)	54 (60.0%)	684 (41.5%)	133 (48.5%)
No labour	137 (7.1%)	124 (7.0%)	13 (9.0%)	4 (13.8%)	126 (7%)	7 (7.8%)	123 (7.5%)	14 (5.1%)
Mode of delivery n (%)								
Elective cesarean section	93 (4.8%)	84 (4.7%)	9 (6.2%)	3 (10.3%)	84 (4.7%)	6 (6.7%)	83 (5.0%)	10 (3.6%)
Emergency cesarean section	288 (15.0%)	261 (14.7%)	27 (18.6%)	1 (3.4%)	265 (14.7%)	22 (24.4%)	244 (14.8%)	44 (16.1%)
Instrumental	326 (17.0%)	304 (17.1%)	22 (15.2%)	4 (13.8%)	311 (17.3%)	11 (12.2%)	278 (16.9%)	48 (17.5%)
Vaginal	1214 (63.2%)	1127 (63.5%)	87 (60.0%)	21 (72.4%)	1142 (63.4%)	51 (56.7%)	1042 (63.3%)	172 (62.8%)
Birth weight in grams,	3200	3200	3200	3030	3200	3500	3210	3080
median (IQR)	(2900, 3500)	(2910, 3500)	(2790, 3510)	(2600, 3220)	(2900, 3500)	(3220, 3770)	(2920, 3520)	(2760, 3360)
Birth weight percentile	31.5	31.7	29.8	15.9	31.5	61.9	33.7	20.5
Median (IQR)	(12.6, 59.0)	(12.9, 58.8)	(9.75, 59.8)	(2.85, 45.6)	(12.6, 58.8)	(30.2, 79.1)	(14.0, 61.0)	(6.72, 42.1)
Birth weight <10th percentile, n (%)	413 (21.5%)	375 (21.1%)	38 (26.2%)	14 (48.3%)	385 (21.4%)	14 (15.6%)	326 (19.8%)	87 (31.8%)
Birth weight <3rd percentile, n (%)	186 (9.7%)	170 (9.6%)	16 (11.0%)	9 (31.0%)	174 (9.7%)	3 (3.3%)	143 (8.7%)	43 (15.7%)
Birth weight >95th percentile, n (%)	38 (2.0%)	34 (1.9%)	4 (2.8%)	0 (0%)	31 (1.7%)	7 (7.8%)	37 (2.2%)	1 (0.4%)
Developed preeclampsia n(%)	82 (4.3%)	69 (3.9%)	13 (9.0%)	1 (3.4%)	73(4.1%)	8 (8.9%)	73 (4.4%)	9 (3.3%)
Developed pregnancy hypertension n (%)	43 (2.2%)	38 (2.1%)	5 (3.4%)	0	36 (2.0%)	7 (7.8%)	36 (2.2%)	7 (2.6%)
Developed gestational diabetes n (%)	455 (23.7%)	406 (22.9%)	49 (33.8%)	3 (10.3%)	408 (22.6%)	44 (48.9%)	386 (23.4%)	69 (25.2%)

Results are expressed as the median (interquartile rage, IQR) and n and percentage (%) as required.

**Table 3:** Summary results from multiple logistic regression analyses.

Pregnancy Complication	Maternal Age ≥ 40 (n = 145)		BMI < 18 (n = 29)		BMI ≥ 35 (n = 90)		Smoking (n = 274)	
	aOR (95% CI)	*p*	aOR (95% CI)	*p*	aOR (95% CI)	*p*	aOR (95% CI)	*p*
Preterm delivery	0.99 (0.45 to 1.96)	0.970	2.67 (0.77 to 7.13)	0.077	1.01 (0.38 to 2.26)	0.978	0.78 (0.42 to 1.36)	0.415
Cesarean section	1.13 (0.70 to 1.79)	0.612	0.71 (0.20 to 1.92)	0.539	2.12 (1.25 to 3.54)	0.005	0.97 (0.68 to 1.36)	0.851
Vaginal delivery	0.87 (0.57 to 1.32)	0.504	1.35 (0.60 to 3.27)	0.478	0.63 (0.38 to 1.03)	0.064	1.04 (0.78 to 1.40)	0.786
Birth weight <10th percentile	1.54 (1.00 to 2.37)	0.049	3.28 (1.51 to 7.05)	0.002	0.73 (0.40 to 1.34)	0.308	1.83 (1.36 to 2.46)	<0.001
Birth weight <3rd percentile	1.19 (0.63 to 2.11)	0.569	3.73 (1.54 to 8.37)	0.002	0.31 (0.10 to 1.02)	0.055	1.91 (1.29 to 2.78)	0.001
Birth weight >95th percentile	1.31 (0.44 to 3.89)	0.623	1.19 (0.07 to 18.84)	0.908	3.50 (1.37 to 8.91)	0.009	0.15 (0.01 to 0.70)	0.061
Preeclampsia	2.00 (0.91 to 4.11)	0.070	0.97 (0.05 to 4.85)	0.977	1.94 (0.76 to 4.31)	0.129	0.79 (0.35 to 1.56)	0.522
Gestational diabetes mellitus	1.61 (1.08 to 2.36)	0.018	0.32 (0.05 to 1.07)	0.118	3.10 (1.95 to 4.89)	<0.001	1.06 (0.76 to 1.45)	0.745

BMI: body mass index; aOR: adjusted odds ratio (the complete models are provided in the Supplementary Materials); CI: confidence index; *p*: *p*-value. Highlighted in bold *p* < 0.05.

## Data Availability

The data presented in this study are available upon request from the corresponding author and under the condition of approval from the relevant Research Ethics Committees due to data protection regulations.

## Supplementary Materials

The following supporting information can be downloaded at: https://www.mdpi.com/article/10.3390/medicina60071071/s1.
