# Supplementary material for: Association between Perinatal Outcomes and Maternal Risk Factors: A Cohort Study
Source: Medicina (Kaunas). 2024 Jun 29;60(7):1071. doi: 10.3390/medicina60071071 (PMC11278671; doi:10.3390/medicina60071071)
Supplement: Supplementary file 1 [file medicina-60-01071-s001.zip › medicina-3037942-supplementary.pdf]

Table S1: Association between maternal age > 40 and preeclampsia, preterm delivery (>37 w), gestational diabetes, fetal growth, and mode of delivery

	Peeeclamspia			*Preterm delivery (<37w)*			*GDM*			*Fetal growth <10thcentile*			*Fetal growth <3rd centile*			*C to Section*			*Eutocic Delivery*		
Predictors	OR	95% CI	p	OR	95% CI	p	OR	95% CI	p	OR	95% CI	p	OR	95% CI	p	OR	95% CI	p	OR	95% CI	p
Intercept	0.00	0.00 to 0.01	< 0.001	0.07	0.02 to 0.20	< 0.001	0.02	0.01 to 0.03	< 0.001	0.31	0.15 to 0.60	0.001	0.54	0.20 to 1.48	0.225	0.02	0.01 to 0.05	< 0.001	14.74	8.27 to 26.54	< 0.001
Age <=40		Reference			Reference			Reference			Reference			Reference			Reference			Reference	
Age >40	2.00	0.91 to 4.11	0.070	0.99	0.45 to 1.96	0.970	1.61	1.08 to 2.36	0.018	1.54	1.00 to 2.37	0.049	1.19	0.63 to 2.11	0.569	1.13	0.70 to 1.79	0.611	0.87	0.57 to 1.32	0.504
BMI	1.07	1.03 to 1.12	0.001	0.99	0.95 to 1.02	0.475	1.10	1.08 to 1.13	< 0.001	0.96	0.93 to 0.98	0.001	0.91	0.87 to 0.94	< 0.001	1.04	1.02 to 1.07	< 0.001	0.97	0.95 to 0.99	0.002
Smoker	0.78	0.35 to 1.55	0.514	0.78	0.42 to 1.36	0.411	1.02	0.73 to 1.40	0.905	1.78	1.33 to 2.39	< 0.001	1.88	1.27 to 2.73	0.001	0.94	0.66 to 1.32	0.728	1.08	0.81 to 1.45	0.608
Previous CS																					
Parous no CS		-			-			-			-			-			Reference			Reference	
Nulliparous																3.97	2.92 to 5.45	<0.001	0.15	0.12 to 0.20	<0.001
Previous *CS*																14.01	9.72 to 20.40	<0.001	0.09	0.06 to 0.12	0.001
Previous BW<P3																					
Parous no BW<P3		-			-			-			-			Reference			-			-	
Nulliparous													2.11	1.48 to 3.05	<0.001						
Previous *BW<P3*													5.24	3.16 to 8.62	< 0.001						
Previous BW<P10																					
Parous no BW<P10		-			-			-			Reference			-			-			-	
Nulliparous										3.20	2.42 to 4.22	<0.001									
Previous *BW<P10*										4.20	2.97 to 5.94	< 0.001									
Previous GDM																					
Parous no GDM		-			-			Reference			-			-			-			-	
Nulliparous							1.31	1.03 to 1.66	0.026												
Previous GDM							4.91	2.80 to 8.72	<0.001												
Previous PB																					
Parous no PB		-			Reference			-			-			-			-			-	
Nulliparous				1.46	0.97 to 2.22	0.070															
Previous PB				3.34	1.58 to 6.62	0.001															
Previous PE																					
Parous no PE		Reference			Reference			-			Reference			Reference			-			-	
Nulliparous	3.10	1.82 to 5.49	< 0.001																		
Previous PE	8.91	3.57 to 21.34	< 0.001	1.05	0.34 to 2.71	0.930				0.66	0.29 to 1.51	0.322	0.70	0.20 to 1.89	0.522						
cHT	10.37	4.19 to 25.39	< 0.001	4.79	1.7 to 12.16	0.002	2.73	1.19 to 6.33	0.017	2.65	1.09 to 6.45	0.032	4.82	1.50 to 13.21	0.004	1.71	0.67 to 4.15	0.242	0.95	0.40 to 2.31	0.906
Aspirin <16w	1.28	0.53 to 2.83	0.564		-			-			-			-			-			-	
Conception																					
Natural		Reference			Reference			-			Reference			Reference			Reference			Reference	
IVF	1.84	0.75 to 4.10	0.156	1.33	0.55 to 2.82	0.492				0.80	0.47 to 1.34	0.393	1.02	0.48 to 1.98	0.956	2.15	1.32 to 3.45	0.002	0.61	0.38 to 0.96	0.034
Ovulation D	1.04	0.06 to 5.18	0.970	3.06	0.87 to 8.43	0.048				1.79	0.76 to 4.21	0.181	2.22	0.71 to 5.84	0.129	0.63	0.18 to 1.74	0.418	1.41	0.62 to 3.33	0.419
Family PE	1.64	0.63 to 3.70	0.271		-			-			-			-			-			-	
Previous BW>P90		-			-		1.97	1.10 to 3.45	0.019		-			-			-			-	
GDM	3.12	0.44 to 13.28	0.172	1.96	0.29 to 7.64	0.395		-		0.66	0.14 to 3.18	0.606		-		2.83	0.82 to 9.03	0.084	0.44	0.14 to 1.41	0.165

BMI: body mass index, CS: cesarean section, BW: birth weight, GDM: gestational diabetes mellitus, PE: preeclampsia, IVF: in vitro fertilization.

Table S2: Association between BMI < 18 and preeclampsia, preterm delivery (>37 w), gestational diabetes, fetal growth, and mode of delivery

	Peeeclampsia			*Preterm delivery (<37w)*			gestational diabetes mellitus			Fetal growth <10thcentile			Fetal growth <3rd centile		C to Section			Eutocic Delivery	
Predictors	OR	95% CI	p	OR	95% CI	p	OR	95% CI	p	OR	95% CI	p	OR	95% CI	OR	95% CI	p	OR	95% CI
Intercept	0.01	0.00 to 0.06	< 0.001	0.04	0.01 to 0.16	< 0.001	0.03	0.01 to 0.08	0.001	0.03	0.01 to 0.07	< 0.001	0.03	0.01 to 0.09	0.02	0.01 to 0.06	< 0.001	23.48	10.67 to 52.50
BMI>= 18	Reference			Reference			Reference			Reference			Reference		Reference			Reference	
BMI< 18	0.97	0.05 to 4.85	0.977	2.67	0.77 to 7.13	0.077	0.32	0.05 to 1.07	0.118	3.28	1.51 to 7.05	0.002	3.73	1.54 to 8.37	0.71	0.20 to 1.92	0.539	1.35	0.60 to 3.27
Maternal age	1.01	0.96 to 1.07	0.573	1.01	0.97 to 1.05	0.808	1.06	1.03 to 1.08	<0.001	1.04	1.01 to 1.06	0.004	1.02	0.98 to 1.05	1.04	1.01 to 1.07	0.007	0.96	0.94 to 0.98
Smoker	0.79	0.36 to 1.57	0.536	0.77	0.41 to 1.33	0.375	1.10	0.79 to 1.49	0.573	1.80	1.34 to 2.41	< 0.001	1.84	1.25 to 2.67	0.97	0.68 to 1.37	0.877	1.04	0.77 to 1.39
Previous CS	-			-			-			-			-						
Parous no CS															Reference			Reference	
Nulliparous															4.19	3.07 to 5.80	<0.001	0.14	0.11 to 0.18
Previous CS															14.10	9.79 to 20.52	0.001	0.08	0.06 to 0.12
Previous BW <P3	-			-			-			-					-			-	
Parous no BWt<P3													Reference						
Nulliparous													2.33	1.62 to 3.38					
Previous *BWt<P3*													5.58	3.37 to 9.15					
Previous BW<P10	-			-			-						-		-			-	
Parous no BW<P10										Reference									
Nulliparous										3.55	2.68 to 4.74	<0.001							
Previous BW *<P10*										4.37	3.10 to 6.18	<0.001							
Previous GDM	-			-						-			-		-			-	
Parous no GDM							Reference												
Nulliparous							1.37	1.09 to 1.74	0.008										
Previous GDM							5.38	3.13 to 9.39	< 0.001										
Previous PB	-						-			-			-		-			-	
Parous no PB				Reference															
Nulliparous				1.49	0.98 to 2.28	0.065													
Previous PB				3.43	1.62 to 6.80	0.001													
Previous PE							-								-			-	
Parous no PE	Reference			Reference						Reference			Reference						
Nulliparous	2.85	1.65 to 5.06	< 0.001																
Previous PE	8.67	3.43 to 21.00	< 0.001	1.03	0.34 to 2.68	0.948				0.69	0.28 to 1.50	0.379	0.73	0.21 to 1.96					
Cronic hipertension	14.90	6.04 to 36.22	< 0.001	4.46	1.62 to 10.99	0.002	4.09	1.86 to 9.16	<0.001	2.12	0.85 to 4.95	0.091	3.05	0.98 to 7.93	1.98	0.79 to 4.78	0.134	0.86	0.36 to 2.09
Aspirin <16w	1.24	0.52 to 2.74	0.617	-			-			-			-		-			-	
Conception							-												
Natural	Reference			Reference						Reference			Reference		Reference			Reference	
IVF	2.17	0.91 to 4.78	0.064	1.32	0.55 to 2.80	0.506				0.76	0.44 to 1.26	0.300	0.99	0.47 to 1.92	1.75	1.07 to 2.81	0.023	0.75	0.47 to 1.18
Ovulation drugs	0.94	0.05 to 4.71	0.953	3.10	0.88 to 8.58	0.046				1.68	0.69 to 3.85	0.232	2.18	0.70 to 5.60	0.58	0.16 to 1.59	0.335	1.55	0.68 to 3.68
Family PE	1.71	0.66 to 3.85	0.228	-			-			-			-		-			-	
PreviousBW >P90	-			-			2.26	1.28 to 3.90	0.004	-			-		-			-	
GDM	2.81	0.36 to 12.85	0.241	2.00	0.30 to 7.72	0.381	-			0.68	0.10 to 2.66	0.626	-		2.83	0.81 to 9.19	0.089	0.43	0.13 to 1.41

BMI: body mass index, CS: cesarean section, BW: birth weight, GDM: gestational diabetes mellitus, PE: preeclampsia, IVF: in vitro fertilization.

Table S3: Association between BMI > 40 and preeclampsia, preterm delivery (>37 w), gestational diabetes, fetal growth, and mode of delivery

	Peeeclamspia			*Preterm delivery (<37w)*			*Gestational Diabetes Mellitus*			*Fetal growth <10thcentile*			*Fetal growth <3rd centile*			*Cesarean*			*Eutocic Delivery*		
Predictors	OR	95% CI	p	OR	95% CI	p	OR	95% CI	p	OR	95% CI	p	OR	95% CI	p	OR	95% CI	p	OR	95% CI	p
Intercept	0.01	0.00 to 0.06	0.001	0.04	0.01 to 0.18	< 0.001	0.03	0.01 to 0.06	<0.001	0.04	0.02 to 0.09	<0.001	0.04	0.01 to 0.12	<0.001	0.02	0.01 to 0.05	<0.001	25.36	11.56 to 56.56	<0.001
BMI < 35		Reference			Reference			Reference			Reference			Reference			Reference			Reference	
BMI>= 35	1.94	0.76 to 4.31	0.129	1.01	0.38 to 2.26	0.978	3.10	1.95 to 4.89	<0.001	0.73	0.40 to 1.34	0.308	0.31	0.10 to 1.02	0.055	2.12	1.25 to 3.54	0.005	0.63	0.38 to 1.03	0.064
Maternal age	1.02	0.97 to 1.07	0.541	1.00	0.96 to 1.04	0.939	1.06	1.04 to 1.09	<0.001	1.03	1.01 to 1.06	0.010	1.01	0.98 to 1.05	0.484	1.04	1.01 to 1.07	0.005	0.96	0.94 to 0.98	<0.001
Smoker	0.80	0.36 to 1.58	0.547	0.78	0.42 to 1.36	0.409	1.08	0.78 to 1.48	0.629	1.81	1.35 to 2.43	<0.001	1.88	1.28 to 2.74	0.001	0.97	0.68 to 1.37	0.877	1.04	0.78 to 1.40	0.795
Previous CS																					
Parous no CS		-			-			-			-			-			Reference			Reference	
Nulliparous																4.28	3.13 to 5.93	<0.001	0.14	0.11 to 0.18	<0.001
Previous CS																14.19	9.84 to 20.67	<0.001	0.08	0.06 to 0.12	<0.001
Previous BW <P3																					
Parous no BWt<P3		-			-			-			-			Reference			-			-	
Nulliparous													2.29	1.58 to 3.31	<0.001						
Previous *BWt<P3*													5.62	3.42 to 9.24	<0.001						
Previous BW<P10																					
Parous no BW<P10		-			-			-			Reference			-			-			-	
Nulliparous										3.52	2.65 to 4.67	<0.001									
Previous BW *<P10*										4.35	3.08 to 6.15	<0.001									
Previous GDM																					
Parous no GDM		-			-			Reference			-			-			-			-	
Nulliparous							1.40	1.10 to 1.78	0.005												
Previous GDM							5.06	2.92 to 8.91	<0.001												
Previous PB																					
Parous no PB		-			Reference			-			-			-			-			-	
Nulliparous				1.49	0.98 to 2.28	0.065															
Previous PB				3.39	1.61 to 6.72	0.001															
Previous PE																					
Parous no PE		Reference			Reference			-			Reference			Reference			-			-	
Nulliparous	2.90	1.68 to 5.16	< 0.001																		
Previous PE	8.62	3.42 to 20.81	< 0.001	1.05	0.34 to 2.71	0.922				0.69	0.30 to 1.56	0.370	0.72	0.24 to 2.14	0.550						
Cronic hipertension	13.30	5.37 to 32.57	< 0.001	4.38	1.57 to 10.96	0.003	3.49	1.55 to 7.95	0.002	2.19	0.91 to 5.28	0.080	3.49	1.23 to 9.91	0.019	1.73	0.67 to 4.23	0.238	0.93	0.39 to 2.29	0.881
Aspirin <16w	1.30	0.54 to 2.86	0.539		-			-			-			-			-			-	
Conception																					
Natural		Reference			Reference			-			Reference			Reference			Reference			Reference	
IVF	2.19	0.92 to 4.83	0.061	1.31	0.55 to 2.79	0.512				0.75	0.45 to 1.26	0.281	0.97	0.49 to 1.95	0.937	1.78	1.09 to 2.86	0.019	0.74	0.46 to 1.17	0.204
Ovulation drugs	0.94	0.05 to 4.70	0.955	3.04	0.86 to 8.40	0.050				1.65	0.71 to 3.87	0.247	2.16	0.78 to 6.00	0.141	0.56	0.16 to 1.55	0.310	1.57	0.68 to 3.71	0.293
Family PE	1.69	0.66 to 3.79	0.227		-			-			-			-			-			-	
PreviousBW >P90		-			-		2.20	1.24 to 3.83	0.006		-			-			-			-	
GDM	2.87	0.38 to 12.92	0.227	1.96	0.29 to 7.58	0.392		-		0.66	0.14 to 3.14	0.604		-		2.85	0.82 to 9.15	0.085	0.44	0.13 to 1.40	0.162

BMI: body mass index, CS: cesarean section, BW: birth weight, GDM: gestational diabetes mellitus, PE: preeclampsia, IVF: in vitro fertilization.

Table S4: Association between smoking and preeclampsia, preterm delivery (>37 w), gestational diabetes, fetal growth, and mode of delivery.

	Peeeclamspia			*Preterm delivery (<37w)*			*Gestational Diabetes Mellitus*			*Fetal growth <10thcentile*			*Fetal growth <3rd centile*			*Cesarean*			*Eutocic Delivery*		
Predictors	OR	95% CI	p	OR	95% CI	p	OR	95% CI	p	OR	95% CI	p	OR	95% CI	p	OR	95% CI	p	OR	95% CI	p
Intercept	0.00	0.00 to 0.01	< 0.001	0.06	0.01 to 0.35	0.002	0.00	0.00 to 0.01	< 0.001	0.10	0.03 to 0.29	< 0.001	0.31	0.07 to 1.35	0.119	0.01	0.00 to 0.02	< 0.001	55.78	21.53 to 147.20	< 0.001
Not smoker		Reference			Reference			Reference			Reference			Reference			Reference			Reference	
Smoker	0.79	0.35 to 1.56	0.522	0.78	0.42 to 1.36	0.415	1.06	0.76 to 1.45	0.745	1.83	1.36 to 2.46	< 0.001	1.91	1.29 to 2.78	0.001	0.97	0.68 to 1.36	0.851	1.04	0.78 to 1.40	0.786
Maternal age	1.01	0.96 to 1.07	0.598	1.00	0.96 to 1.04	0.929	1.06	1.04 to 1.09	< 0.001	1.03	1.01 to 1.06	0.007	1.02	0.98 to 1.05	0.310	1.04	1.01 to 1.07	0.006	0.96	0.94 to 0.98	< 0.001
Body Mass index	1.08	1.03 to 1.12	< 0.001	0.99	0.95 to 1.02	0.474	1.11	1.08 to 1.13	< 0.001	0.96	0.93 to 0.98	0.001	0.91	0.87 to 0.94	< 0.001	1.04	1.02 to 1.07	< 0.001	0.97	0.95 to 0.99	0.002
Previous <u>CS</u>																					
Parous no CS		-			-			-			-			-			Reference			Reference	
Nulliparous																4.32	3.16 to 5.98	<0.001	0.14	0.11 to 0.18	<0.001
Previous CS																13.87	9.61 to 20.21	< 0.001	0.09	0.06 to 0.12	< 0.001
Previous BW <P3																					
Parous no BWt<P3		-			-			-			-			Reference			-			-	
Nulliparous													2.20	1.52 to 3.20	<0.001						
Previous *BWt<P3*													5.21	3.14 to 8.57	<0.001						
Previous BW<P10																					
Parous no BW<P10		-			-			-			Reference			-			-			-	
Nulliparous										3.43	2.59 to 4.58	<0.001									
Previous BW *<P10*										4.19	2.97 to 5.93	<0.001									
Previous GDM																					
Parous no GDM		-			-			Reference			-			-			-			-	
Nulliparous							1.45	1.14 to 1.84	0.003												
Previous GDM							4.71	2.68 to 8.40	< 0.001												
Previous PB																					
Parous no PB		-			Reference			-			-			-			-			-	
Nulliparous				1.47	0.96 to 2.25	0.074															
Previous PB				3.34	1.58 to 6.61	0.001															
Previous PE																					
Parous no PE		Reference			Reference			-			Reference			Reference			-			-	
Nulliparous	3.04	1.76 to 5.42	< 0.001																		
Previous PE	8.55	3.40 to 20.63	< 0.001	1.05	0.34 to 2.72	0.924				0.67	0.27 to 1.47	0.353	0.70	0.20 to 1.92	0.536						
Cronic hipertension	10.84	4.36 to 26.64	< 0.001	4.75	1.69 to 12.06	0.002	2.56	1.11 to 5.98	0.027	2.62	1.04 to 6.21	0.033	4.77	1.48 to 13.04	0.004	1.59	0.62 to 3.87	0.319	1.03	0.42 to 2.51	0.956
Aspirin <16w	1.31	0.54 to 2.91	0.531		-			-			-			-			-			-	
Conception																					
Natural		Reference			Reference			-			Reference			Reference			Reference			Reference	
IVF	2.22	0.92 to 4.92	0.060	1.30	0.54 to 2.78	0.518				0.75	0.44 to 1.24	0.274	0.97	0.46 to 1.89	0.938	1.78	1.09 to 2.86	0.020	0.74	0.46 to 1.18	0.205
Ovulation drugs	0.97	0.05 to 4.86	0.978	3.04	0.86 to 8.43	0.050				1.64	0.67 to 3.81	0.254	2.14	0.68 to 5.62	0.150	0.58	0.16 to 1.60	0.342	1.55	0.68 to 3.66	0.306
Family PE	1.59	0.62 to 3.58	0.294		-			-			-			-			-			-	
PreviousBW >P90		-			-		1.94	1.08 to 3.41	0.023		-			-			-			-	
GDM	3.13	0.43 to 13.43	0.176	1.96	0.29 to 7.63	0.395		-		0.67	0.10 to 2.66	0.615		-		2.83	0.82 to 9.01	0.085	0.44	0.14 to 1.42	0.168

BMI: body mass index, CS: cesarean section, BW: birth weight, GDM: gestational diabetes mellitus, PE: preeclampsia, IVF: in vitro fertilization.
